# Susceptibility and resistance of Gram-negative bacteria to a novel antimicrobial agent TGV-49

**DOI:** 10.3389/frabi.2025.1615821

**Published:** 2025-11-03

**Authors:** Victor V. Tetz, Kristina M. Kardava, Maria F. Vecherkovskaya, Semen A. Leyn, Marinela L. Elane, Andrei L. Osterman, George V. Tetz

**Affiliations:** ^1^ Department of Systems Biology, Human Microbiology Institute, New York, NY, United States; ^2^ Sanford Burnham Prebys Medical Discovery Institute, La Jolla, CA, United States; ^3^ Tetz Laboratories, New York, NY, United States

**Keywords:** antibiotic resistance, TGV-49, experimental evolution, gram–negative bacteria, *Acinetobacter baumannii*, ESKAPE, morbidostat

## Abstract

**Introduction:**

Antimicrobial resistance remains a major global public health challenge that necessitates novel drugs with a low resistance rate.

**Methods:**

Herein, we evaluate TGV-49, a novel broad-spectrum antimicrobial agent, against multidrug-resistant Gram-negative bacteria, including ESKAPE pathogens (*Acinetobacter baumannii*, *Pseudomonas aeruginosa*, and *Klebsiella pneumoniae*) and pathogens from agriculture that infect humans (*Ralstonia solanacearum* and *Aeromonas hydrophila*).

**Results:**

TGV-49 was highly effective in overcoming resistance to conventional antibiotics. The experimental evolution of *A. baumannii* using a morbidostat revealed minimal development of resistance.

**Conclusion:**

Our findings suggest TGV-49 as a potential alternative for combating MDR infections in clinical and agricultural settings.

## 1 Introduction

Antimicrobial resistance (AMR) remains among the most challenging global public health threats. Over 4.71 million deaths reported in 2021 were associated with bacterial AMR, including over 1.1 million deaths directly attributable to bacterial AMR (Naghavi et al., 2024). Among bacteria with the highest contribution to the AMR burden, Gram-negative pathogens, including *Burkholderia cepacia* complex, *Serratia marcescens* spp., and representatives of the ESKAPE group *Enterobacter* spp., *Klebsiella pneumoniae*, *Acinetobacter baumannii*, and *Pseudomonas aeruginosa*, pose a significant healthcare risk (Exner et al., 2017; Cristina et al., 2019; Dacco et al., 2023; Halawa et al., 2024; Miller and Arias, 2024).

ESKAPE pathogens are the primary causative agents for lower respiratory and bloodstream infections, the carbapenem-resistant mutants of which are associated with significant morbidity and mortality rates of <70% in certain cases (Tumbarello et al., 2015; Tamma et al., 2024; De Oliveira et al., 2020; Bassetti and Garau, 2021).

While ESKAPE pathogens are primarily associated with healthcare settings, another group of Gram-negative pathogens that poses an evolving threat to human health are plant and aquaculture primary pathogens (Li et al., 2024; Verweij et al., 2022; Rooney et al., 2020; Piotrowska and Popowska, 2014). These pathogens cause infections in immunocompromised individuals and act as reservoirs for antibiotic cross-resistance to related human pathogenic bacterial species within the pangenome (Reynolds and Kollef, 2021).

Effective antibiotic therapy faces several limitations, leading to a shortage of viable treatment options for human infections caused by Gram-negative bacteria (Dan and Talapan, 2024). One of the primary limitations is the intrinsic resistance of Gram-negative bacteria owing to multiple or pan-resistance mechanisms, rapid acquisition of resistance through gene transfer and mutations, and biofilm formation, spreading antibiotic resistance to healthcare pathogens from agriculture and aquaculture (Gerba, 2009; Li et al., 2024). The lack of efficient methods for antibiotic selection in healthcare settings is another major challenge (Manyi-Loh et al., 2018; Iwu et al., 2020; Tetz and Tetz, 2022a; Tetz et al., 2023b). Finally, not all antibiotic resistance mechanisms have been elucidated, making it difficult to fully understand and resolve this problem. In recent years, several previously unknown regulatory pathways contributing to bacterial resistance have been discovered, changing the paradigms on how AMR is established and maintained in the bacterial population.

Among them is the newly described Universal Receptive System formed by the extracellular DNA- and RNA-based Teazeled receptors on the outer surface of prokaryotic and eukaryotic cells, and includes the work of integrases and recombinases (Tetz and Tetz, 2022b; Tetz et al., 2025a). In bacteria, the Universal Receptive System regulates interactions between cells and the outer environment, including responses to antimicrobial agents on genetic and epigenetic levels. It also orchestrates the formation and maintenance of the cell memory (Tetz and Tetz, 2022b; Tetz et al., 2025a, 2025b).

Therefore, understanding the genetic mechanisms driving the development of AMR in Gram-negative pathogens is critical for developing novel drugs with a low resistance rate. The morbidostat device is one of the most sophisticated approaches for investigating resistance development in laboratory environments. It integrates experimental evolution through continuous culturing cycles with genome sequencing of the evolving isolates and characterization of the drug-resistant isolates produced (Leyn et al., 2021). The morbidostat can identify various resistance mechanisms based on specific genetic mutations in the presence of different concentrations of the tested antimicrobial agent (Leyn et al., 2021).

Our group previously developed Mul-1867, a novel antimicrobial agent exhibiting broad-spectrum antimicrobial activity by attacking the microbial cell wall. Mul-1867 is highly effective against resistant clinical bacterial and fungal isolates, as well as pre-formed biofilms (Tetz and Tetz, 2015; Tetz et al., 2016). Its derivative TGV-28 has high activity against plant pathogens (Kardava et al., 2023; Tetz et al., 2023a).

In the present study, we investigated another derivative of Mul-1867 developed by our group, TGV-49 (Tetz Group Variant-49, poly-N1-hydrazino(imino)methyl-1,6-hexanediamine) against Gram-negative pathogens, including those from the ESKAPE group (Tetz and Tetz, 2015; Tetz et al., 2016). TGV-49 shares the exact mechanism of action against bacterial cells; its positively charged hexanediamine groups bind to negatively charged bacterial membrane components (e.g., phospholipids, fatty acids), while the hydrazine groups react with carbonyl groups, thereby disrupting the microbial membrane.

This dual interaction leads to disruption of the cell wall/membrane, rapid leakage of intracellular contents (evidenced by DNA release), and eventual cell lysis. Electron microscopy confirmed that treated bacteria exhibit membrane damage and collapse (Tetz and Tetz, 2015).

TGV-49 was selected over other tested derivatives due to its higher specific activity against Gram-negative pathogens. We also used a morbidostat-based resistomics workflow to elucidate the mechanism underlying the development of resistance to TGV-49 in *Acinetobacter baumannii*, one of the most difficult Gram-negative pathogens to treat (Magnet et al., 2001; Zhang et al., 2024).

## 2 Materials and methods

### 2.1 Bacterial strains and growth conditions

The bacterial strains *A. baumannii* (ATCC BAA-1605, ATCC BAA-1710, and ATCC 17978)*, K. pneumoniae* (ATCC BAA-1705), *B. cenocepacia* (ATCC-25416), and *P. aeruginosa* (ATCC BAA-2108) were purchased from the American Type Culture Collection (Manassas, VA, USA). Bacterial and fungal clinical isolates *K. pneumoniae* (VT-2646)*, K. oxytoca* (VT-8943)*, B. cenocepacia* (VT-2613)*, P. aeruginosa* (VT*-*7530, VT-2824)*, P. fluorescens* (VT-2535), *Enterobacter cloacae* (VT-1667, VT-4918)*, S. marcescens* (VT-3904)*, Aeromonas hydrophila* (VT-1409)*, Flavobacterium columnare* (VT-5506)*, Ralstonia solanacearum* (VT-5012)*, Xanthomonas campestris* (VT-4657)*, Erwinia amylovora*, (VT-3286), and *Agrobacterium tumefaciens* (VT-2679) were obtained from a private collection provided by Human Microbiology Institute (NY, USA).

{/it}All specimens were microbial suspensions derived from isolated colonies cultivated on agar plates using Columbia Blood Agar Base (Oxoid, ThermoFisher, USA). The suspensions were incubated in Mueller–Hinton broth (MHB) (Oxoid, ThermoFisher, USA) to guarantee uniform growth conditions for subsequent experimental analysis.

### 2.2 Antimicrobial susceptibility testing

The MICs were determined using the broth macrodilution method, as described by the Clinical and Laboratory Standards Institute M27-A3 (CLSI) (Clinical and Laboratory Standards Institute, 2019). The MIC was defined as the lowest concentration of the antimicrobial agent that inhibits bacterial growth and possesses an optical density (OD570) increase ≤ 0.05 under cultivation conditions (Dong et al., 2012). Bacteria were cultivated in Mueller-Hinton broth at 37°C, except for *Flavobacterium columnare*, which was cultivated at 25°C for 24 h.

Resistance or sensitivity to antibiotics was evaluated based on the breakpoints established by the CLSI and European Committee on Antimicrobial Susceptibility Testing (EUCAST) guidelines, and it was dependent on the antibiotic used and the pathogen tested (CLSI, 2019, 2022).

The following antimicrobial agents were used in the study: Ampicillin, Amoxicillin, Piperacillin, Ceftriaxone, Ceftazidime, Cefepime, Aztreonam, Imipenem, Meropenem, Fosfomycin, Gentamicin, Tobramycin, Amikacin, Ciprofloxacin, Levofloxacin, Polymyxin B, Colistin, Doxycycline, Tetracycline, Chloramphenicol, Trimethoprim, Sulfamethoxazole, and Rifaximin (all from Sigma-Aldrich).

**Figure 1:**
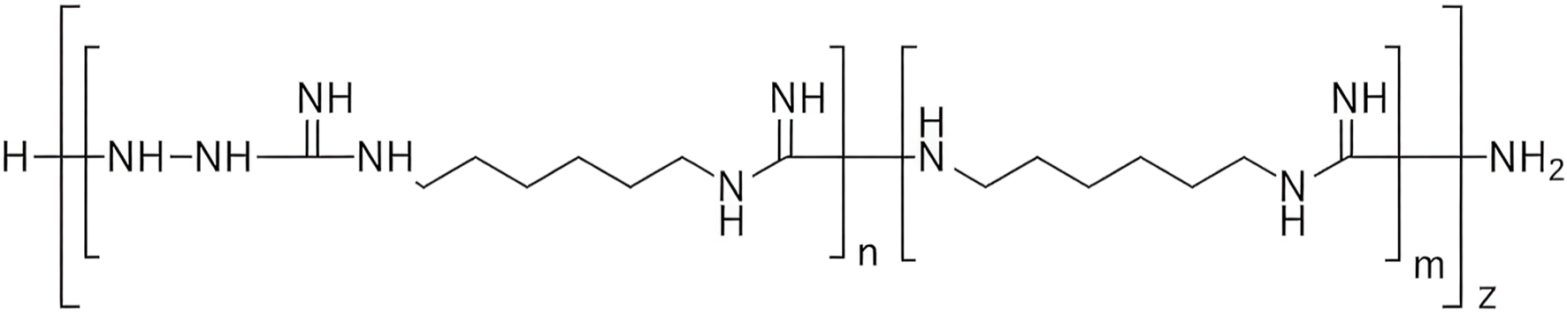
Chemical structure of TGV-49.

TGV-49 (Human Microbiology Institute) is a derivative of Mul-1867 with more polymeric chains, resulting in a higher level of antimicrobial activity (**Figure 1**). For each study, the control samples were cultivated under the same conditions in antibiotic-free medium.

### 2.4 Experimental evolution in the morbidostat and clonal analysis

For the experimental evolution under TGV-49 pressure, we used the morbidostat device, custom-engineered based on general principles introduced by Toprak et al. (2013). Briefly, the morbidostat is a computer-controlled chemostat-like continuous culturing bioreactor where density of bacterial culture, which is constantly monitored by OD600, is controlled by varying an antibiotic concentration in media. Automated dilutions switch between addition of; (i) drug-containing media (increase of a drug concentration) when bacterial cultures are growing faster than dilution rate, and (ii) drug-free media (decrease of a drug concentration) when the growth rate slows down due to excessive drug pressure. This algorithm enables a gradually growing selective pressure driving the evolution of higher drug resistance. The detailed description of morbidostat implementation is provided on GitHub (https://github.com/sleyn/morbidostat_construction). Morbidostat-based experimental evolution of resistance to TGV-49 for *A. baumannii* ATCC 17978 was performed as previously described (Zlamal et al., 2021). Briefly, a morbidostat run was performed for 5 days upon inoculation of all six glass reactors with six individually prepared log-phase cultures derived from glycerol stocks of six individual colonies of *A. baumannii* ATCC17978 (Zlamal et al., 2021) at starting OD600 = 0.02 (20 mL in MHB/DMSO). During the run, the TGV-49 concentration in the drug-containing feed bottle was 200 mg/L (approximately 32xMIC of the unevolved parental strain). Glass reactor tubes were replaced every ~24 h (10 mL samples were taken) to minimize the impact of a biofilm accumulating on the walls of each reactor. For each reactor, glycerol stocks were prepared for all samples (taken daily) (B–F), and individual clones were randomly selected upon plating an aliquot of sample F on MHB-agar (15 clones from each reactor).

The obtained 90 clones were profiled for acquired resistance by growing them in 96-well microtiter plates in liquid MHB media without drug and supplemented with TGV-49 at 20 and 40 mg/L (corresponding to 1x and 2x MIC of unevolved strain, respectively). Clones with confirmed acquired resistance (≥ 2xMIC) were selected for the Illumina-based whole genome sequencing (WGS).

Genomic DNA was extracted using the GenElute bacterial genomic DNA kit (Sigma-Aldrich) using the manufacturer’s NA2110 protocol for Gram-negative bacteria. Libraries for sequencing were prepared using a NEBNext Ultra II FS DNA library prep kit for Illumina modules E7810L and E7595L (New England BioLabs) as per the manufacturer’s protocol (DNA input  ≥100 ng); TruSeq DNA PCR-free CD index 20015949 (Illumina) adapters were used to eliminate PCR amplification. Size selection and cleanup were performed using magnetic beads AMPure XP (Beckman Coulter). Prepared libraries were quantified using a NEBNext Library Quant kit for Illumina E7630L (New England BioLabs) and pooled with volumes adjusted to normalize concentrations for ∼200-fold genomic coverage. Pooled library size and quality were analyzed with the 2100 Bioanalyzer instrument (Agilent). Pooled DNA libraries were sequenced by Novogene Co. using HiSeq 4000 (*A. baumannii* clones) or HiSeq X10 (all other samples) instruments (Illumina) with paired-end 150-bp read length. Our computational pipeline used for WGS data analysis and variant calling has been previously described (Zlamal et al., 2021).

### 2.5 Statistical analyses

Data were analyzed using GraphPad Prism version 9.3.1. Two-way analysis of variance (ANOVA) was used for multiple comparisons at the 95% confidence level (*p* < 0.05). Data are presented as the mean ± standard deviation (SD) with three independent replicates. All experiments were performed in triplicate in three independent experiments.

## 3 Results

### 3.1 Susceptibility and broad-spectrum activity of TGV-49 against human, animal, and plant pathogens

**Figure 2:**
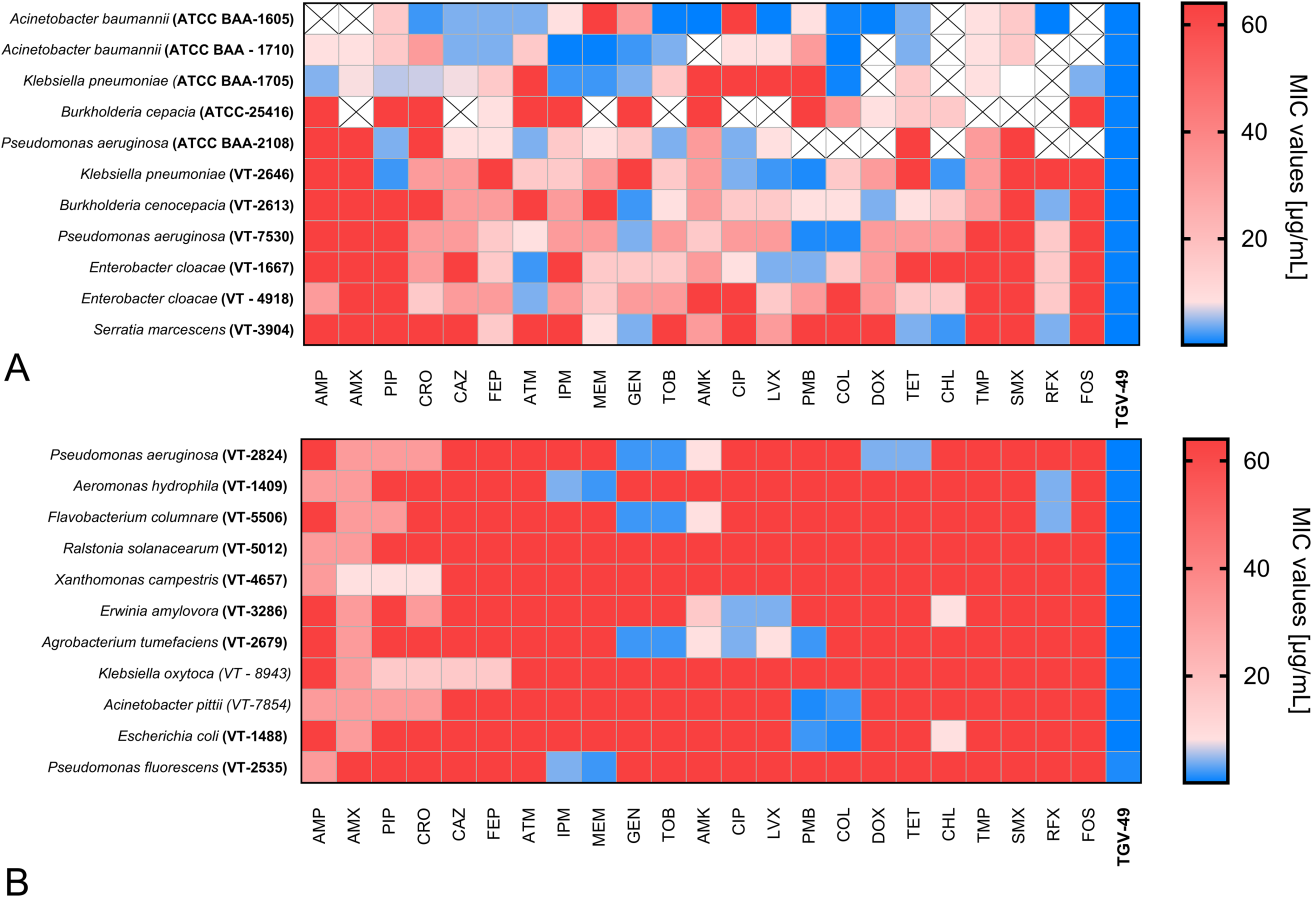
Antibiotic susceptibility of Gram-negative bacteria to TGV-49 and conventional antibiotics. The heatmap illustrates the antibiotic susceptibility of various Gram-negative bacterial **(A)** human pathogens and **(B)** plant and fish pathogens. The antibiotics tested against the particular bacterial strain are listed at the bottom of the heatmap. The color range reflects the MIC values (µg/mL); blue represents lower MIC values, while red represents higher MIC values (corresponding to higher resistance). The “cross” states that a particular pathogen was not tested against that particular antibiotic. Antibiotic AMP, Ampicillin; AMX, Amoxicillin; PIP, Piperacillin; CRO, Ceftriaxone; CAZ, Ceftazidime; FEP, Cefepime; ATM, Aztreonam; IPM, Imipenem; MEM, Meropenem; FOS, Fosfomycin; GEN, Gentamicin; TOB, Tobramycin; AMK, Amikacin; CIP, Ciprofloxacin; LVX, Levofloxacin; PMB, Polymyxin B; COL, Colistin; DOX, Doxycycline; TET, Tetracycline; CHL, Chloramphenicol; TMP, Trimethoprim; SMX, Sulfamethoxazole; RIF, Rifaximin.

The antimicrobial susceptibility testing demonstrated the potent broad-spectrum activity of TGV-49 against various Gram-negative bacterial strains. Notably, all tested multidrug-resistant clinical isolates (*Acinetobacter baumannii, Klebsiella pneumoniae, Burkholderia cepacia, Pseudomonas aeruginosa*, and *Enterobacter cloacae*) were resistant to a wide range of antibiotics, including beta-lactams, aminoglycosides, carbapenems, macrolides, and quinolones (**Figure 2A**). Some of the tested strains exhibited resistance even to last-resort treatments such as colistin. However, all these strains, including carbapenem-resistant Gram-negative bacilli from ESKAPE pathogens, were highly susceptible to TGV-49, with minimum inhibitory concentrations (MIC) ranging from 0.06 to 4.0 μg/mL.

Similarly, the antimicrobial testing of plant and fish pathogens (*P. aeruginosa, P. fluorescens, A. hydrophila, A. tumerafaciens, F. columnare, R. solanacearum, X. campestris*, and *E. amylovora*) demonstrated significant resistance to various common antibiotics used in humans (**Figure 2B**). However, these pathogens were susceptible to TGV-49 with low MIC values, comparable with those for human pathogens. The list of antibiotics used in this study and the results of susceptibility testing are presented in **Figures 2A, B**.

### 3.2 Experimental evolution of TGV-49 resistance in *A. baumannii ATCC 17978*

To investigate the dynamics of TGV-49 resistance development, we employed a morbidostat-based comparative resistomics workflow, as described by Zlamal et al. (2021), for clinically relevant Gram-negative bacteria, *Acinetobacter baumannii* ATCC 17978. Experimental evolution was performed in 6 parallel, 20 mL glass reactors of the custom-designed continuous culturing device (morbidostat), as described by Leyn et al. (2021). The procedure was performed under increasing pressure of TGV-49 drug at a concentration range of 0–200 mg/L as a function of growth rate assessed by constant monitoring of optical density (OD600). Five days of experimental evolution were sufficient for *A. baumannii* to acquire robust resistance against all tested clinical and experimental antibiotics (Zlamal et al., 2021; Leyn et al., 2023; Zampaloni et al., 2024). During this period, we observed no appreciable trend toward resistance against TGV-49. This observation was confirmed by MIC profiling of randomly picked clones (15 clones from each of the 6 reactors). Indeed, a mild resistance (2-4xMIC) was detected only among the clones from one (R5) of the six reactors.

We selected five clones (5F1–5F5, isolated from sample F taken from Reactor 5 close to the end of the 5-day evolutionary run) for analysis performed using Illumina-based whole-genomic sequencing (WGS). Data processing was performed by our computational pipeline (Zlamal et al., 2021), yielding and annotating Single Nucleotide Variants (SNV), short indels, Copy Number Variants (CNV), and insertion of IS-elements using the iJump algorithm (Zlamal et al., 2021). The results are listed in **Supplementary Table S1A-C**, and the significant variants are summarized in **Supplementary Table S1D**. All clones harbored essentially the same set of variants, including (i) SNV in the tRNA-Arg-CCG, (ii) IS-insert upstream of the *pgsA* gene, which encodes phospholipid biosynthesis enzyme (CDP-diacylglycerol–glycerol-3-phosphate 3-phosphatidyltransferase), and (iii) ~5–10-fold amplification of two genomic loci (24.3 and 13.5 Kb). Of these, the most likely hypothetical candidate for driver event is the amplification of the second locus, which contains three paralogous genes adeT1, adeT2, and adeT3 encoding a putative RND-type efflux pump; this has previously been implicated in aminoglycoside resistance (Magnet et al., 2001).

While the identity of the actual drug target(s) and resistance driver gene(s) (and, thus, the mechanisms of action and resistance) remain elusive, with the hypothesized impact of AdeT-driven efflux being a subject of prospective genetic exploration, this study points to a very low propensity of *A. baumannii* to acquire TGV-49 resistance.

## 4 Discussion

This study underscores the broad-spectrum antimicrobial activity of TGV-49 against a wide range of Gram-negative pathogens, including those linked to human, animal, and plant health.

TGV-49 (poly-N1-hydrazino(imino)methyl-1,6-hexanediamine) has a non-specific action against bacterial cells by targeting negatively charged bacterial membrane components and disrupting the microbial membrane. Among human pathogens, TGV-49 was highly active against Gram-negative representatives of the ESKAPE group, including pathogens harboring resistance to beta-lactams, aminoglycosides, quinolones, and, most clinically important, carbapenem-resistant strains. These organisms are often implicated in severe hospital-acquired infections, particularly in immunocompromised patients with limited treatment options (Lehman and Grabowicz, 2019; Banneman and Rana, 2020; Reynolds and Kolef, 2021; Kunz Coyne et al., 2022). The demonstrated susceptibility of these strains to TGV-49 presents a promising alternative for managing difficult-to-treat infections. Given TGV-49’s activity against various multidrug-resistant pathogens, we focused on the development of resistance to TGV-49. For this purpose, we used a morbidostat system to study the evolution of resistance in *A. baumannii* to TGV-49. In these settings, *A. baumannii* showed no significant resistance even under continuous selective pressure, with only mild resistance emerging in a small subset of bacterial clones. This finding is particularly important as it suggests that TGV-49 has a lower tendency to promote rapid resistance evolution than many current antibiotics, which often become ineffective due to the swift development of resistant bacterial strains.

In conclusion, this study demonstrated the effectiveness of TGV-49 against human pathogens and its activity against agricultural and aquacultural pathogens. As antibiotic resistance continues to pose a global public health threat, TGV-49 provides a promising alternative for topical therapies to existing drugs, with the added benefit of minimizing the risk of resistance development. The broad applicability across diverse pathogen groups necessitates further research into the potential uses of TGV-49 and its derivatives.

## Data Availability

The DNAseq data files have been deposited into bioProject, Accession ID PRJNA1280665. Bacterial strains from the Human Microbiology Institute are available upon request from George Tetz lab (info@hmi-us.com).
