# Supplementary material for: Susceptibility and resistance of Gram-negative bacteria to a novel antimicrobial agent TGV-49
Source: Front Antibiot. 2025 Nov 3;4:1615821. doi: 10.3389/frabi.2025.1615821 (PMC12620978; doi:10.3389/frabi.2025.1615821)
Supplement: Supplementary file 2 [file Table2.docx]

**Supplementary Table 2.** **Examples of morbidostat-based evolution of drug resistance in Gram-negative bacterial pathogens**
